# Endosulfine-alpha inhibits membrane-induced α-synuclein aggregation and protects against α-synuclein neurotoxicity

**DOI:** 10.1186/s40478-016-0403-7

**Published:** 2017-01-10

**Authors:** Daniel Ysselstein, Benjamin Dehay, Isabel M. Costantino, George P. McCabe, Matthew P. Frosch, Julia M. George, Erwan Bezard, Jean-Christophe Rochet

**Affiliations:** 1 Department of Medicinal Chemistry and Molecular Pharmacology, Purdue University, West Lafayette, IN USA; 2 Purdue Institute for Integrative Neuroscience, Purdue University, West Lafayette, IN USA; 3 Université de Bordeaux, Institut des Maladies Neurodégénératives, UMR 5293 Bordeaux, France; 4 CNRS, Institut des Maladies Neurodégénératives, UMR 5293 Bordeaux, France; 5 Department of Neurology, Massachusetts Alzheimer’s Disease Research Center, Massachusetts General Hospital, Charlestown, MA USA; 6 Department of Statistics, Purdue University, West Lafayette, IN USA; 7 Department of Biological and Experimental Psychology, School of Biological and Chemical Sciences, Queen Mary University of London, London, UK

**Keywords:** α-Synuclein, Endosulfine-alpha, Neurodegeneration, Parkinson’s disease, Synucleinopathy

## Abstract

Neuropathological and genetic findings suggest that the presynaptic protein α-synuclein (aSyn) is involved in the pathogenesis of synucleinopathy disorders, including Parkinson’s disease (PD), dementia with Lewy bodies (DLB) and multiple system atrophy. Evidence suggests that the self-assembly of aSyn conformers bound to phospholipid membranes in an aggregation-prone state plays a key role in aSyn neurotoxicity. Accordingly, we hypothesized that protein binding partners of lipid-associated aSyn could inhibit the formation of toxic aSyn oligomers at membrane surfaces. To address this hypothesis, we characterized the protein endosulfine-alpha (ENSA), previously shown to interact selectively with membrane-bound aSyn, in terms of its effects on the membrane-induced aggregation and neurotoxicity of two familial aSyn mutants, A30P and G51D. We found that wild-type ENSA, but not the non-aSyn-binding S109E variant, interfered with membrane-induced aSyn self-assembly, aSyn-mediated vesicle disruption and aSyn neurotoxicity. Immunoblotting analyses revealed that ENSA was down-regulated in the brains of synucleinopathy patients versus non-diseased individuals. Collectively, these results suggest that ENSA can alleviate neurotoxic effects of membrane-bound aSyn via an apparent chaperone-like activity at the membrane surface, and a decrease in ENSA expression may contribute to aSyn neuropathology in synucleinopathy disorders. More generally, our findings suggest that promoting interactions between lipid-bound, amyloidogenic proteins and their binding partners is a viable strategy to alleviate cytotoxicity in a range of protein misfolding disorders.

**Electronic supplementary material:**

The online version of this article (doi:10.1186/s40478-016-0403-7) contains supplementary material, which is available to authorized users.

## Introduction

The presence of cytoplasmic inclusions containing aggregated forms of the presynaptic protein α-synuclein (aSyn) is characteristic of a panel of neurodegenerative diseases commonly referred to as synucleinopathies, including Parkinson’s disease (PD), dementia with Lewy bodies (DLB) and multiple system atrophy [49]. PD is further characterized by a loss of dopaminergic neurons in the *substantia nigra* region of the brain. Autosomal dominant mutations in the SNCA gene encoding aSyn, including substitutions (A30P, E46K, H50Q, G51D, A53E, and A53T) and gene multiplications, have been linked to familial forms of PD [42, 43, 48]. Some aSyn substitution mutants have an increased propensity to aggregate into soluble oligomers or insoluble fibrils [12], and gene multiplications are predicted to promote aSyn aggregation via mass action. Accordingly, it is hypothesized that preventing the initiation of aSyn aggregation could be neuroprotective in PD and other synucleinopathies.

aSyn is most commonly expressed as a 14 kDa (140-residue) protein. Evidence suggests that the protein exists as a natively unfolded monomer in solution [56] and as a compact disordered monomer in mammalian cells [51], although other findings suggest that it can also adopt multimeric structures in the cytoplasm [5]. The protein consists of 3 regions: an N-terminal region containing a series of lysine-rich repeats, a central hydrophobic region, and a C-terminal region enriched with proline residues and acidic residues. aSyn interacts with anionic phospholipid vesicles by forming an amphipathic α-helix (most likely an α11/3 helix) with various lengths, ranging from a short helix spanning residues ~1–25 to a long helix spanning residues ~1–97 and including the central hydrophobic region [4, 6, 9, 25], and this interaction is thought to be necessary for the protein’s function in regulating synaptic vesicle trafficking [13, 16, 53].

aSyn has been shown to undergo accelerated aggregation at membrane surfaces when incubated with synthetic or natural phospholipid vesicles [20, 27, 36] or supported lipid bilayers [22, 41], presumably because the two dimensional surface of the membrane increases the probability of molecular interactions needed for oligomerization [1]. Membrane-induced aSyn aggregation is likely driven by a disruption of contacts between the central hydrophobic domain and the membrane, resulting in the exposure of hydrophobic residues that can then engage in interactions involved in aSyn self-assembly [6, 58]. Evidence suggests that aSyn aggregation on the membrane plays a key role in neurotoxicity [58], potentially by triggering membrane thinning, a process that could result in increased ion permeability [11, 37, 44].

Based on evidence that membrane-induced aSyn aggregation plays a key role in aSyn-mediated neurodegeneration, we hypothesize that proteins that interact with membrane-bound aSyn can interfere with the formation of neurotoxic aSyn oligomers at the membrane surface. This inhibitory effect could involve a shift in the equilibrium of membrane-bound aSyn species in favor of a less exposed state or a disruption of interactions between neighboring exposed aSyn conformers on the bilayer. To address this hypothesis, we examined the effects of the protein endosulfine-alpha (ENSA) on membrane-induced aSyn aggregation and aSyn neurotoxicity. ENSA, a 13 kDa protein that is a member of the cAMP-regulated phosphoprotein family, has a defined role in cell cycle regulation in various tissues but is also expressed in post-mitotic neurons in the CNS [17]. Although the role of ENSA in the CNS is poorly understood, the protein has been found to be significantly down-regulated in Alzheimer’s disease (AD) and in Down syndrome [30, 31]. Wild-type (WT) ENSA, but not a variant with the S109E phosphomimetic substitution, was shown to bind specifically to membrane-associated aSyn, but not aSyn in the absence of phospholipids [7, 57]. Here, we characterized WT ENSA and S109E in terms of their impact on membrane-induced aggregation, vesicle permeabilization, and dopaminergic cell death elicited by the familial aSyn mutants, A30P and G51D. Our results provide strong support for the idea that aSyn aggregation at membrane surfaces plays a key role in aSyn neurotoxicity, and they suggest that stabilization of membrane-bound aSyn could be a therapeutic strategy for PD and other synucleinopathy disorders.

## Materials and methods

### Materials

Unless otherwise stated, all chemicals were purchased from Sigma-Aldrich (St. Louis, MO). Isopropyl β-D-1-thiogalactopyranoside (IPTG), ampicillin, and phenylmethylsulfonyl fluoride (PMSF) were purchased from Gold Biotechnology (St. Louis, MO). L-α-phosphatidylcholine (egg PC), L-α-phosphatidylglycerol (egg PG), and phospholipid extrusion membranes were purchased from Avanti Polar Lipids (Alabaster, AL). Dulbecco’s minimal essential media (DMEM), penicillin-streptomycin, the ViraPower Adenoviral Expression System, and LR clonase II were obtained from Invitrogen (Carlsbad, CA). Fetal bovine serum (FBS) was obtained from Corning Life Sciences (Corning, NY). Iodixanol and ECF substrate were obtained from GE Life Sciences (Pittsburgh, PA). 100 kDa spin filters were purchased from Millipore (Billerica, MA). Bicinchoninic acid (BCA) assay reagents and Coomassie Brilliant Blue were purchased from Thermo Scientific (Rockford, IL).

### Antibodies

The following antibodies were used in these studies: mouse anti-aSyn (#610787, BD Biosciences, San Jose, CA); rabbit anti-ENSA (#D5Z1U), rabbit anti-β3-tubulin (#D65A4), and AP-linked anti-mouse and anti-rabbit IgG (Cell Signaling Technology, Danvers, MA); chicken anti-MAP2 (#CPCA-MAP2, EnCor Biotechnology, Gainesville, FL); rabbit anti-TH (#AB152, Millipore, Billerica, MA); mouse anti-β-actin (#A5316 or #A5441, Sigma-Aldrich); and anti-rabbit IgG-Alexa Fluor 488 and anti-chicken IgG-Alexa Fluor 594 (Invitrogen, Carlsbad, CA).

### Protein purification

Recombinant aSyn was purified from BL21 (DE3) cells transformed with pT7-7 constructs encoding A30P or G51D as described [58]. A cDNA encoding S109E was generated via overlap-extension PCR using a pET21-ENSA construct as the DNA template and cloned as an NdeI-BamHI fragment into the pT7-7 expression vector. Protein expression was induced by adding IPTG (final concentration, 1 mM) to an *E. coli* culture pre-grown to an OD_600_ of 0.6, and the culture was incubated at 37 °C for 3 h. The cells were harvested by centrifugation, suspended in a buffer consisting of 10 mM Tris HCl, pH 7.4, 1 mM PMSF, and 1 mM EDTA, and lysed with a French pressure cell (psi 1000). After lysis, 0.1% (w/v) streptomycin sulfate was added to the lysate, which was then cleared by centrifugation. ENSA was partially purified from the resulting supernatant by ammonium sulfate precipitation (30% saturation). The pellet was re-suspended in 10 mM Tris HCl, pH 7.4, and loaded on a DEAE-Sepharose anion exchange column. The column flow-through was analyzed by SDS-PAGE, and fractions containing ENSA were loaded onto a Sephacryl S-100 column. Fractions enriched with ENSA to a purity of >98%, as determined by SDS-PAGE with Coomassie Blue staining, were pooled, concentrated, aliquoted, and stored at −80 °C until use.

### Lipid vesicle preparation

Small unilamellar vesicles (SUVs) were prepared as described [58]. Egg PG and egg PC suspended in chloroform were mixed at an equimolar ratio in a round bottom flask. The chloroform was evaporated under a nitrogen stream and further dried under vacuum for 1 h. Dried lipids were suspended in PBS (10 mM phosphate buffer, 2.7 mM KCl, and 137 mM NaCl, pH 7.4), and 50 nm SUVs were prepared by extruding the suspension through a Whatman membrane. The size of the vesicles was confirmed by dynamic light scattering. Lipid vesicles were stored at 4 °C prior to use.

### Lipid flotation assay

Lipid flotation analyses were carried out as described [58]. aSyn was dialyzed overnight into PBS with 0.02% (w/v) NaN_3_, and the solution was filtered by successive centrifugation steps through a 0.22 μm spin filter and a 100 kDa centrifugal filter to isolate monomeric protein. aSyn (40 μM) was incubated with PG:PC SUVs (protein-to-lipid ratio, 1:20 mol/mol) in the absence or presence of ENSA (10–40 μM) at 37 °C for 72 h in a total volume of 60 μL. After incubation, the sample was mixed with 4 mL of 30% (v/v) iodixanol solution and overlaid with 7.0 mL of 25% (v/v) iodixanol and 350 μL of 5% (v/v) iodixanol in a polyalomar tube (Beckman, Miami, FL). All of the above iodixanol solutions were prepared in lipid flotation buffer (10 mM HEPES, pH 7.4, 150 mM NaCl). The samples were spun at 200,000 × g in a Beckman SW 41 Ti rotor for 4 h. The membrane fraction was carefully collected from the 5% iodixanol fraction at the top of the gradient, concentrated using a 10 kDa spin filter, and stored at −20 °C prior to Western blot analysis.

Membrane-bound proteins isolated by lipid flotation were analyzed by Western blotting as described [58]. Protein samples were combined with Laemmli buffer (final concentration, 60 mM Tris HCl, pH 6.8, 2% (w/v) SDS, 10% (v/v) glycerol, 5% (v/v) β-mercaptoethanol, 0.01% (w/v) bromophenol blue) and boiled for 2 min. Denatured proteins were separated via SDS-PAGE on a 4–20% (w/v) polyacrylamide gradient gel (BioRad, Hercules, CA) and transferred to a polyvinylidene difluoride (PVDF) membrane (pore size, 0.4 μm). After blocking in 10% (w/v) non-fat milk, the membrane was washed with PBS + 0.5% Tween 20 (w/v) (PBS-T) and probed for aSyn with the Syn-1 primary antibody, diluted 1/2000 in PBS-T with 1% (w/v) BSA. After washing in PBS-T, the membrane was probed with a secondary anti-mouse antibody conjugated to alkaline phosphatase (diluted 1/3000). To visualize the bands, the membrane was exposed to ECF substrate for 30 s, and images were acquired using a Typhoon imaging system (GE Life Sciences, Pittsburgh, PA). Band densities were quantified using ImageJ software (NIH, Bethesda, MD). Oligomer band intensities were determined by carrying out a densitometric analysis of the region of the gel spanning predicted molecular weights of 30 kDa through 250 kDa. We verified using ImageJ software that the band intensities were below the saturation range. In general, we found that the rate of aSyn aggregation increased in the presence of SUVs that had been stored for ~2-4 weeks at 4 °C [58].

### Membrane disruption assay

Calcein-loaded egg PG:PC SUVs were prepared as described [3] with some modifications. A solution of calcein (170 mM) was prepared in H_2_O using NaOH to adjust the pH to 7.4. The final osmolality was 280 mOsm/kg. The calcein solution was used to resuspend dried egg PG:PC lipids, and the suspension was extruded through a Whatman membrane to generate 50 nm SUVs. Calcein-containing vesicles were isolated from free calcein via gel filtration through a Sephadex G-50 column pre-equilibrated with PBS, pH 7.4, 0.02% (w/v) NaN_3_ (280 mOsm/kg). Fractions containing isolated vesicles were pooled and stored at 4 °C until use. Calcein-loaded vesicles were found to be very stable, with no spontaneous dye leakage observed over several weeks.

For membrane disruption experiments, monomeric aSyn was isolated as described above (see ‘Lipid Flotation Assay’) and dialyzed into PBS with 0.02% (w/v) NaN_3_. aSyn (40 μM) was incubated with calcein-loaded PG:PC SUVs (protein-to-lipid ratio, 1:20 mol/mol) in the absence or presence of ENSA (10–40 μM) at 37 °C in a total volume of 50 μL. At each time point, 3 μL of the reaction mixture was diluted into 180 μL of PBS, and the diluted samples were analyzed with a Fluoromax-3 spectrofluorometer (Horiba Scientific, Edison, New Jersey) (excitation wavelength, 485 nm; emission wavelengths, 505–530 nm; slit width, 1 nm). To determine the maximum dye release, vesicles were lysed by adding 5 μL of 1% (v/v) Triton X-100. The % leakage at time t was determined using the following equation:

$$ \%\  leakage=\frac{I_t-{I}_0}{I_{max}-{I}_0}\times 100\% $$

where I_t_ is the fluorescence emission intensity at time t, I_0_ is the intensity at time 0, and I_max_ is the maximal intensity determined after detergent lysis of the vesicles (intensity values were determined at 515 nm).

### aSyn fibrillization

aSyn fibrillization rates were assessed using a thioflavin T fluorescence assay as described [59], with some modifications. Monomeric aSyn prepared as outlined above (see ‘Lipid Flotation Assay’) was diluted to a final concentration of 40 μM in a solution of PBS, pH 7.4, 0.02% (w/v) NaN_3_, and 20 μM thioflavin T, and the diluted protein solution was aliquoted in triplicate in a 96-well microtiter plate (200 μL per well). A Teflon ball was added to each well to enhance mixing. The plate was incubated at 37 °C with constant shaking in a Genios plate reader (Tecan, Uppsala, Sweden). The formation of amyloid-like fibrils was monitored by measuring thioflavin T fluorescence (excitation wavelength, 440 nm; emission wavelength, 480 nm), with one reading taken every 15 min.

### Preparation of adenoviral constructs

Adenoviruses encoding aSyn and ENSA variants were produced using the ViraPower Adenoviral Expression System. Methods used to generate aSyn-encoding adenoviral DNAs were described previously [58]. A cDNA encoding ENSA was cloned as a KpnI-XhoI fragment into the pENTR1A entry plasmid, and the insert from the pENTR1A construct was transferred into the pAd/CMV/V5 adenoviral expression vector via recombination using Gateway LR Clonase. The sequence of the DNA insert in the resulting adenoviral construct was verified using an Applied Biosystems (ABI3700) DNA sequencer (Purdue Genomics Core Facility).

A low-titer preparation of each adenovirus was produced by lipid-mediated transfection of the HEK 293A packaging cell line with adenoviral DNA. The low-titer preparation was then applied to a new batch of HEK 293A cells to generate an amplified virus preparation. Adenovirus packaged with the plasmid pAd/V5-DEST/LacZ (Invitrogen), encoding the control protein β-galactosidase (β-gal) fused to the V5 epitope, was also prepared to control for non-specific effects of viral transduction.

### Preparation and treatment of primary mesencephalic cultures

Primary midbrain cultures were prepared via dissection of day 17 embryos obtained from pregnant Sprague-Dawley rats (Envigo (Harlan), Indianapolis, IN) using methods approved by the Purdue Animal Care and Use Committee, as described [39, 58]. The mesencephalic region containing the *substantia nigra* and ventral tegmental area was isolated stereoscopically, and the tissue was treated with trypsin (final concentration, 26 μg/mL in 0.9% [w/v] NaCl). Dissociated cells were plated on 96-well plates (pretreated with poly-L-lysine, 5 μg/mL) at a density of 82,000 cells per well in media consisting of DMEM, 10% (v/v) FBS, 10% (v/v) horse serum, penicillin (10 U/mL), and streptomycin (10 μg/mL). Five days after plating, the cells were treated for 48 h with cytosine arabinofuranoside (20 μM) to inhibit the growth of glial cells. At this stage (7 days in vitro), the cultures were transduced for 72 h with adenoviruses encoding aSyn variants at a multiplicity of infection (MOI) of 10, with or without adenovirus encoding ENSA at an MOI of 5. Adenovirus encoding β-gal was used as a control for viral transduction. The cultures were then incubated in fresh media for an additional 24 h, and immunocytochemistry was performed as described [58]. The cells were fixed in 4% (w/v) paraformaldehyde in PBS (pH 7.4) and subsequently permeabilized for 1 h with PBS, pH 7.4, 0.3% (v/v) Triton X-100, 1% (w/v) BSA, and 10% (v/v) FBS. After washing with PBS, the cells were treated for 48 h at 4 °C with chicken anti-MAP2 (1:2000) and rabbit anti-TH (1:500) primary antibodies. The cells were then washed with PBS, treated with AlexaFluor 594-conjugated goat anti-chicken and AlexaFluor 488-conjugated goat anti-rabbit secondary antibodies (both at 1:1000) for 1 h at 22 °C, and washed a final time with PBS before imaging.

### Measurement of neuronal viability

Relative dopaminergic cell viability was assessed by counting MAP2- and TH-immunoreactive primary neurons in a blinded manner [58]. The neurons were imaged using an automated Cytation 3 Cell Imaging Multi-Mode Reader (BioTek, Winooski, VT) equipped with a 4× objective. A total of 9 images were taken to provide representation of the whole well, and at least 900 MAP2^+^ neurons were counted per experiment for each treatment. Each experiment was repeated using cultures prepared from at least 3 different pregnant rats. The data are expressed as a percentage of MAP2^+^ neurons that were also TH^+^ to correct for variations in cell plating density.

### Neurite length measurements

Neurite length measurements were carried out on the same images generated by the Cytation 3 cell imager for neuron viability analysis. Lengths of MAP2^+^ processes extending from TH^+^/MAP2^+^ neurons with an intact cell body (>40 neurons and >70 neurites per experiment for each treatment) were assessed in a blinded manner using the Nikon NIS Elements analysis software (Nikon Instruments, Melville, NY) [58].

### Analysis of brain tissue homogenates

Research involving analyses of coded human brain samples was granted an exemption from requiring ethics approval by the Purdue University and Indiana University Institutional Review Boards. The French brain samples were obtained from brains collected in a Brain Donation Program of the Brain Bank “GIE NeuroCEB” run by a consortium of Patients Associations: ARSEP (association for research on multiple sclerosis), CSC (cerebellar ataxias), France Alzheimer and France Parkinson. The consents were signed by the patients themselves or their next of kin in their name, in accordance with the French Bioethical Laws. The Brain Bank GIE NeuroCEB (Bioresource Research Impact Factor number BB-0033-00011) has been declared at the Ministry of Higher Education and Research and has received approval to distribute samples (agreement AC-2013-1887). Samples of human cortex and *substantia nigra* were resuspended at a concentration of 0.1 mg/mL in RIPA buffer (50 mM Tris HCl, pH 7.4, 150 mM NaCl, 0.1% (w/v) SDS, 0.5% (w/v) sodium deoxycholate, 1% (v/v) Triton X 100) supplemented with protease inhibitor cocktail (Sigma P8340) and PMSF (1 mM). The tissue was homogenized by cup sonication at 4 °C using a Digital Sonifier (Branson, Danbury, CT). The homogenate was cleared by centrifugation at 13,000 × g, and the supernatant was fractionated via SDS-PAGE (20 μg of protein loaded in each lane of a 16% (w/v) polyacrylamide gel) and analyzed via Western blotting (primary antibody: rabbit anti-ENSA, 1:1,000 or 1:2,000; rabbit anti-β3-tubulin, 1:5,000; or mouse anti-β-actin, 1:10,000 (Sigma-Aldrich #A5316) or 1:1,000 (Sigma-Aldrich #A5441); secondary antibody: AP-conjugated anti-rabbit IgG or anti-mouse IgG, 1:3000). ENSA band intensities were determined via densitometric analysis using the ImageJ software and normalized to their respective β-3-tubulin band intensities. In turn, these ratios were normalized to the control ENSA/β-3-tubulin ratio.

### Statistical analysis

Densitometry data from Western blots, calcein dye release data, and primary neuron viability data were analyzed via two-tailed t-test (when comparing 2 groups) or ANOVA followed by Tukey’s multiple comparisons *post hoc* test (when comparing >2 groups) using GraphPad Prism 6.0 (La Jolla, CA). In analyzing percentage dye release data and percentage cell viability data by ANOVA, square root transformations were carried out to conform to ANOVA assumptions. Neurite length data were analyzed using an approach that accounts for (i) the possibility of multiple neurites arising from a single cell, and (ii) comparison across experiments conducted on different days [58]. Neurite lengths for multiple treatment groups were compared using a general linear model implemented in the GLM procedure of SAS Version 9.3 followed by the Tukey’s multiple comparisons post hoc test (Cary, NC).

## Results

### Effect of ENSA on membrane-induced self-assembly of A30P and G51D

The goal of this study was to test the hypothesis that promoting interactions between membrane-associated aSyn and a binding partner at the membrane surface could interfere with lipid-induced aSyn aggregation and aSyn neurotoxicity. To address this hypothesis, we examined the impact of the aSyn-binding protein ENSA on the membrane-induced self-assembly and associated membrane permeabilization activity of toxic aSyn variants. We then sought to determine whether effects of ENSA on membrane-induced aSyn aggregation and aSyn-mediated vesicle disruption in a cell-free system translated into effects on aSyn neurotoxicity in a primary midbrain culture model of PD. Our rationale for characterizing the effects of ENSA was that this protein has been shown to interact with membrane-bound aSyn, whereas it does not bind to aSyn in the absence of phospholipids [57]. Our approach of examining the A30P and G51D familial aSyn mutants was based on our previous finding that both variants bind SUVs with a relatively exposed conformation and have a high propensity to undergo membrane-induced self-assembly and elicit neurotoxicity [58]. In contrast, WT aSyn was not examined here because it has a lower propensity to form aggregates at membrane surfaces and is only weakly neurotoxic in our primary midbrain culture model.

**Fig. 1:**
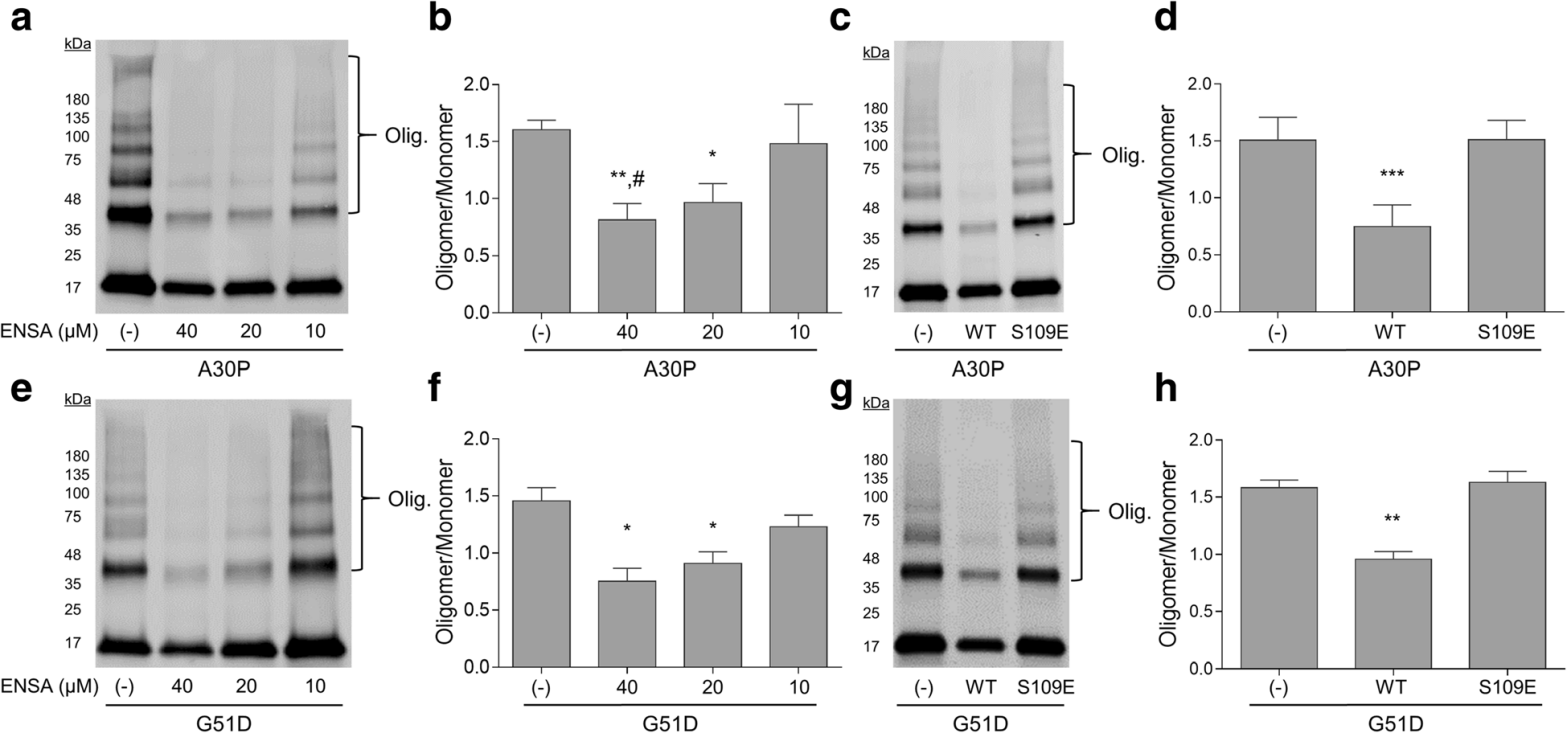
WT ENSA (but not the S109E mutant) inhibits membrane-induced aggregation of A30P and G51D. A mixture of A30P (**a**−**d**) or G51D (**e**−**h**) (40 μM of each) and SUVs was incubated in the absence (−) or presence of WT ENSA (10–40 μM) (**a**, **b**, **e**, **f**) or in the absence (−) or presence of WT ENSA or S109E (40 μM) (**c**, **d**, **g**, **h**) for 72 h. Membrane fractions were isolated and analyzed via Western blotting. (**a**, **c**, **e**, **g**) Representative Western blot images (‘olig’ refers to the region of the blot corresponding to high molecular weight forms of aSyn). (**b**, **d**, **f**, **h**) Bar graphs showing the ratio of total oligomer intensity to monomer intensity determined via densitometric analysis. The data are presented as the mean ± SEM, *n* = 4; *p < 0.05, **p < 0.01, ***p < 0.001 versus control (−); ^#^ *p* < 0.05 versus ‘ENSA, 10 μM’, one-way ANOVA followed by Tukey’s multiple comparisons *post hoc* test

To determine the effect of ENSA on membrane-induced aSyn aggregation, recombinant monomeric A30P or G51D aSyn (40 μM) was incubated with SUVs (800 μM) containing egg PG and egg PC (1:1 mol/mol), in the absence or presence of WT ENSA (10, 20, or 40 μM), at 37 °C for 72 h. We chose to examine PG:PC SUVs because previously we found that this heterogeneous lipid mixture was compatible with the formation of highly stable 50 nm vesicles [58], and SUVs derived from this mixture contain anionic lipids that play a key role in aSyn-membrane interactions [13, 16, 28]. In addition, neuronal membranes (e.g. synaptic vesicle membranes) consist of a mixture of zwitterionic and anionic phospholipids [45, 50]. After the incubation, the membrane fraction was isolated by lipid flotation and analyzed by Western blotting. The lanes containing A30P and G51D incubated in the absence of ENSA displayed pronounced immunoreactive bands at 37, 55 and, 80 kDa and a smearing of immunoreactivity at higher molecular weights, corresponding to SDS-resistant aggregates (Fig. 1a, c, e, g ). Co-incubation of A30P or G51D with ENSA resulted in a decrease in the intensity of the higher molecular weight bands, and densitometric analysis revealed that the magnitude of this decrease was greater for aSyn samples incubated with higher amounts of ENSA (a significant decrease in intensity of ~40 and ~50% was observed for the 20 and 40 μM ENSA treatment groups, respectively) (Fig. 1b, f). In contrast to WT ENSA, the S109E variant previously shown to have a reduced affinity for membrane-bound aSyn [7] had no impact on the formation of high molecular weight species by A30P (Fig. 1c, d) or G51D (Fig. 1g, h) in the presence of SUVs, even when added to the aSyn-lipid mixture at an equimolar level relative to the aSyn protein.

**Fig. 2:**
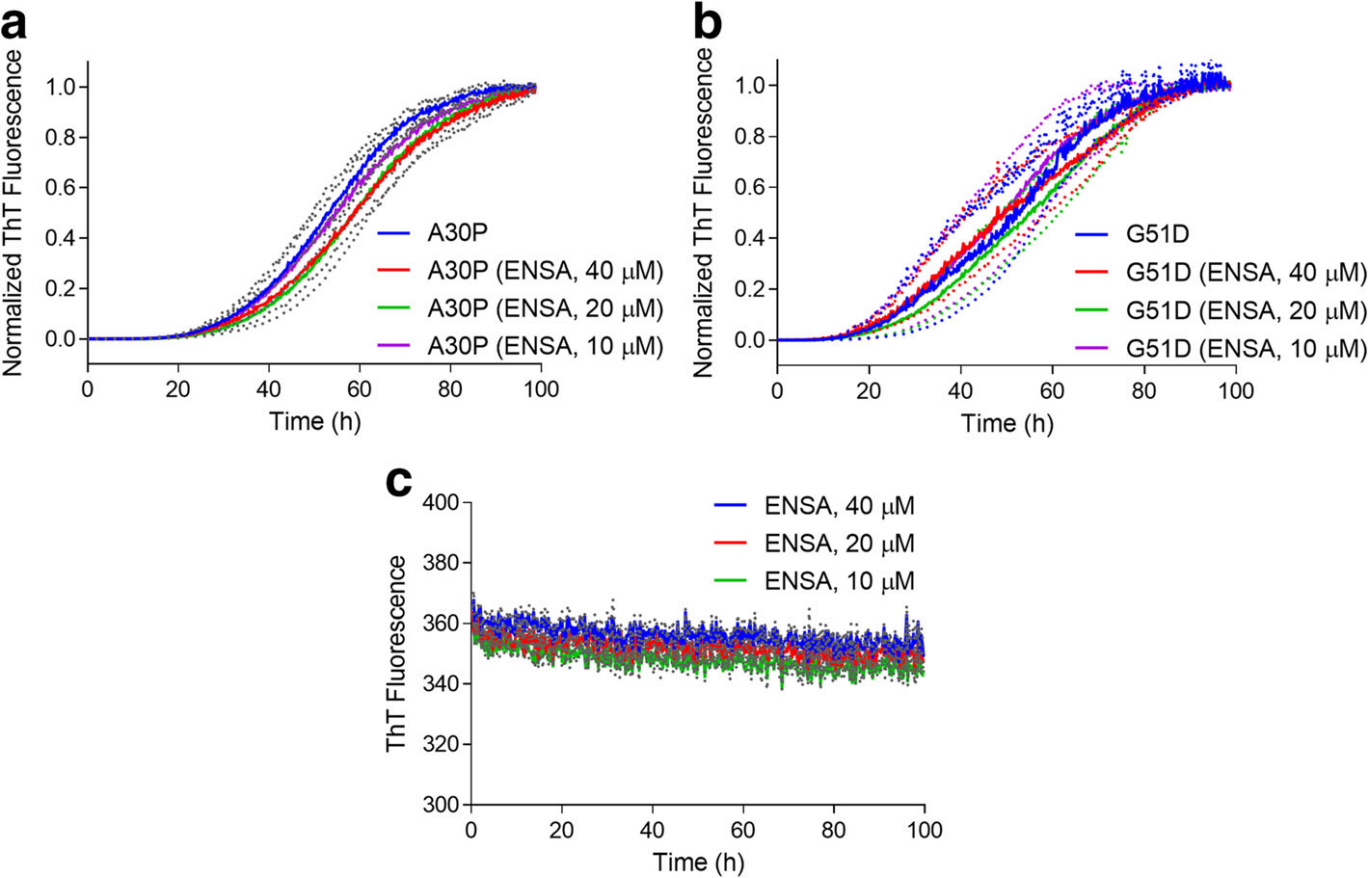
ENSA does not interfere with aSyn fibrillization. The formation of amyloid-like fibrils was monitored in a solution of A30P (**a**) or G51D (**b**) (40 μM of each) with or without ENSA (10–40 μM), or in a solution of ENSA alone (10–40 μM) (**c**). The protein solutions were incubated at 37 °C with constant agitation and analyzed at various times for thioflavin T fluorescence. The graphs show the mean fluorescence normalized to the maximal signal at the end of the incubation (**a**, **b**) or the mean, unnormalized fluorescence (**c**) plotted against the incubation time. The dotted lines above and below each solid line in (**a**) and (**b**) correspond to the mean signal ± SEM; *n* = 3 (**a**), *n* = 4 (**b**), or *n* = 2 (**c**)

We observed that the intensity of the monomer band in the vesicle fraction was greater for A30P or G51D incubated in the absence versus the presence of WT ENSA, or for mutant aSyn incubated in the presence of S109E versus WT ENSA. Similar amounts of membrane-bound aSyn were detected in A30P- or G51D-lipid mixtures incubated with or without WT ENSA for 3 h at 37 °C, conditions permissive for aSyn-lipid binding but not membrane-induced aggregation (Additional file 1: Figure S1). Accordingly, we inferred that the increased monomer immunoreactivity in the membrane fractions of aSyn-lipid mixtures incubated in the absence of ENSA or in the presence of S109E was not due to a greater degree of direct aSyn-vesicle binding, but instead it was a result of the recruitment of unbound monomer from the bulk solution to growing membrane-bound aggregates via a seeding mechanism [58]. In additional experiments, we monitored the formation of amyloid-like fibrils by A30P or G51D in solutions without SUVs using a thioflavin T fluorescence assay. Each aSyn variant was found to undergo fibrillization at similar rates in the absence or presence of WT ENSA (Fig. 2a, b), consistent with the idea that ENSA interacts specifically with membrane-bound aSyn [57]. Importantly, the incubation of ENSA alone did not result in the formation of amyloid-like fibrils (Fig. 2c).

Collectively, these results suggest that (i) WT ENSA interferes with membrane-induced aSyn aggregation; (ii) the inhibitory effect of ENSA on aSyn self-assembly in the presence of SUVs depends strictly on the ability of ENSA to bind aSyn at the membrane surface; and (iii) ENSA has no effect on aSyn fibrillization in the absence of SUVs.

### Effect of ENSA on aSyn-mediated membrane disruption

**Fig. 3:**
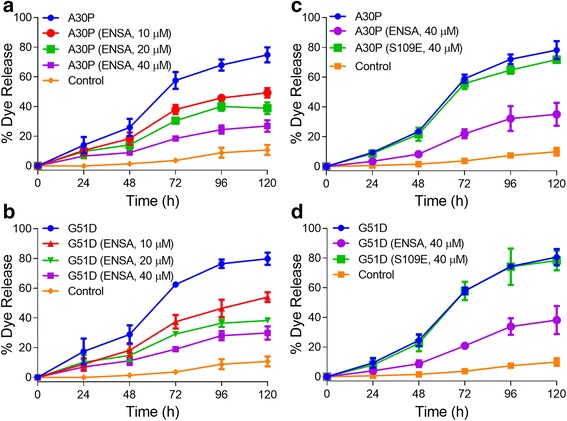
WT ENSA (but not the S109E mutant) interferes with vesicle disruption by A30P and G51D. Calcein-loaded SUVs were incubated in the absence (Control) or presence of A30P (**a**, **c**) or G51D (**b**, **d**) (40 μM of each), with or without WT ENSA (10–40 μM) (**a**, **b**), or with or without WT ENSA or S109E (40 μM) (**c**, **d**). Calcein release was monitored via fluorescence measurements at an excitation wavelength of 485 nm and an emission wavelength of 515 nm. The data are presented as % dye release versus time, with 100% release determined as the signal obtained from vesicles treated with Triton X-100. Mean ± SEM, *n* = 3 or 4. *P* values obtained from a statistical analysis of the data are listed in Additional file1: Tables S1 and S2

Data from several studies suggest that aSyn self-assembly at the membrane surface can promote a disruption of bilayer integrity via a mechanism involving phospholipid extraction by the growing aggregates [37, 44]. Accordingly, we hypothesized that (i) membrane-induced aSyn aggregation should result in vesicle disruption, and (ii) vesicle disruption resulting from membrane-induced aSyn self-assembly should be attenuated by ENSA. To address this hypothesis, we developed a membrane permeabilization assay in which 50 nm PG:PC SUVs pre-loaded with the fluorescent dye calcein at a concentration that resulted in self-quenching were incubated with monomeric aSyn. Vesicle disruption was assessed by monitoring the increase in fluorescence emission resulting from dequenching of the fluorophore upon its release from vesicles and dilution in the surrounding medium. Extended incubation of calcein-loaded SUVs (800 μM) with monomeric A30P or G51D (40 μM) at 37 °C produced a time-dependent increase in fluorescence emission, with a yield of almost 75% of the maximal signal (obtained by lysing the vesicles with the detergent Triton X-100) after 120 h (Fig. 3a-d). Because the non-toxic aSyn variant A29E, previously shown by our group to have a low propensity to undergo membrane-induced aggregation [58], exhibited little measurable activity in this assay (Additional file 1: Figure S2), we inferred that A30P and G51D triggered vesicle disruption via a mechanism related to their aggregation on the membrane surface.

Co-incubation of A30P or G51D with WT ENSA (10, 20, or 40 μM) resulted in a dose-dependent decrease in calcein release (Fig. 3a, b; Additional file 1: Table S1). In contrast, the S109E ENSA variant did not prevent A30P- or G51D-mediated vesicle permeabilization (Fig. 3c, d; Additional file 1: Table S2). Importantly, incubation of ENSA alone with lipid vesicles had no effect on dye release (Additional file 1: Figure S3). Collectively, these data suggested that (i) WT ENSA interferes with the disruption of vesicle integrity resulting from aSyn aggregation at the membrane surface, and (ii) this inhibitory effect depends on the ability of ENSA to bind membrane-associated aSyn.

### Effect of ENSA on aSyn neurotoxicity

**Fig. 4:**
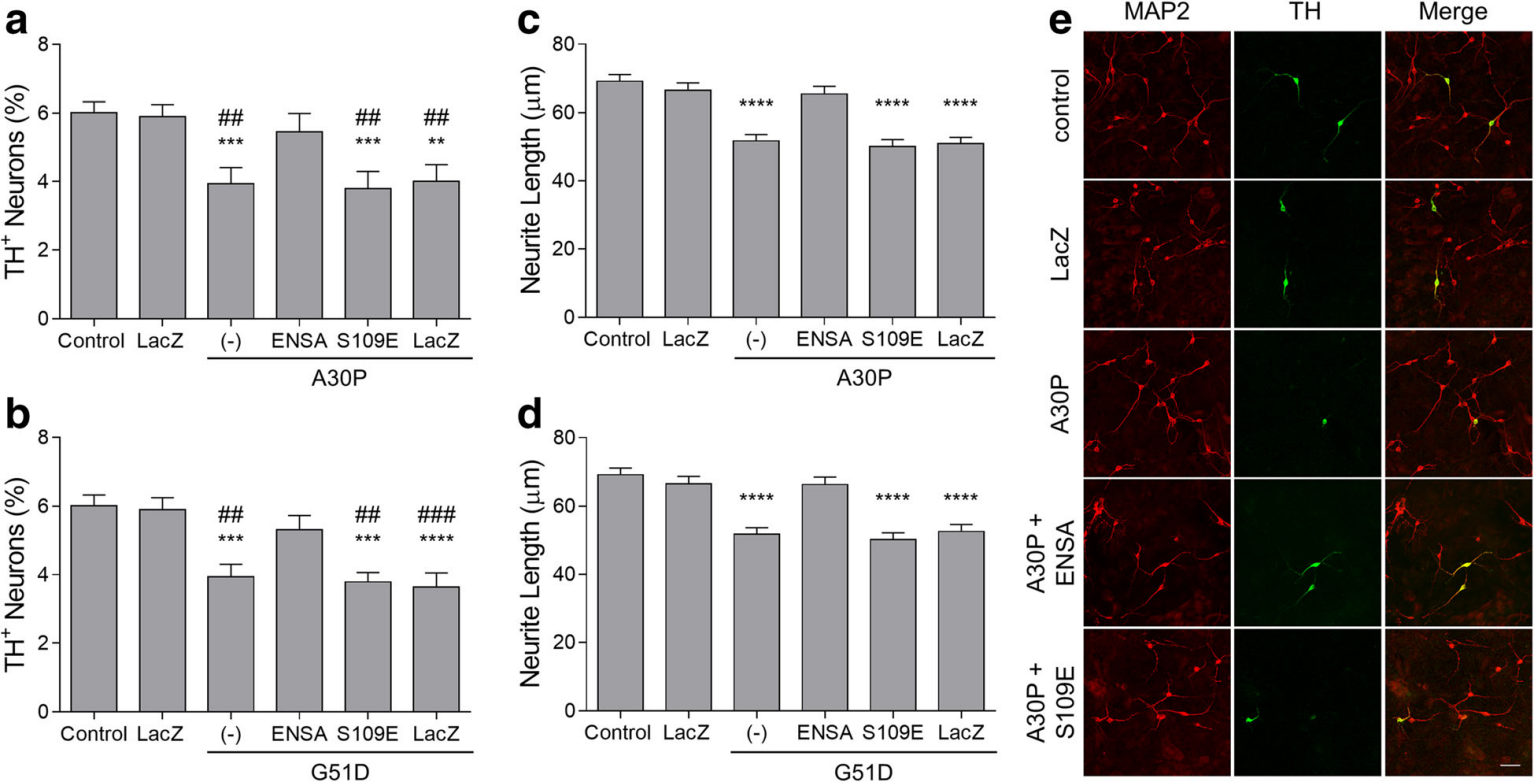
WT ENSA (but not the S109E mutant) alleviates aSyn neurotoxicity. Primary midbrain cultures were transduced with adenovirus encoding A30P (**a**, **c**, **e**) or G51D (**b**, **d**) at an MOI of 10, in the absence or presence of adenovirus encoding WT ENSA, S109E, or the control protein β-gal (LacZ) at an MOI of 5. Additional cultures were untransduced (‘control’) or transduced with LacZ adenovirus alone. The cells were fixed, stained with antibodies specific for MAP2 and TH, and scored for dopaminergic cell viability (**a**, **b**) or neurite lengths (**c**, **d**). The data are presented as the mean ± SEM, *n* = 3. (**a**, **b**) **p < 0.01, ***p < 0.001, ****p < 0.0001 versus control or LacZ, ^##^ *p* < 0.01, ^###^ *p* < 0.001 versus WT ENSA; square root transformation, one-way ANOVA followed by Tukey’s multiple comparisons *post hoc* test. (**c**, **d**) ****p < 0.0001, Tukey’s multiple comparisons *post hoc* test after general linear model implementation. (**e**) Representative fluorescence images showing the more pronounced retraction of neurites extending from MAP2^+^/TH^+^ neurons in cultures expressing A30P alone or A30P with ENSA S109E compared with untransduced cultures, cultures expressing β-gal, or cultures expressing A30P with WT ENSA (scale bar, 50 μm)

Next, we examined whether the ability of ENSA to interfere with membrane-induced aSyn aggregation and aSyn-mediated membrane permeabilization translated into an inhibitory effect of ENSA on aSyn neurotoxicity. To address this question, we characterized ENSA in terms of its ability to alleviate dopaminergic cell death and neurite retraction elicited by mutant aSyn in a primary mesencephalic cell culture model. Cultures were transduced with adenovirus encoding A30P or G51D in the absence or presence of virus encoding WT ENSA, the S109E ENSA variant, or the control protein β-gal. The cells were fixed and stained for microtubule associated protein 2 (MAP2), a general neuronal marker, and tyrosine hydroxylase (TH), a marker of dopaminergic neurons. Relative dopaminergic cell viability was assessed by determining the percentage of MAP2^+^ neurons that were also TH^+^ (previous results indicated that the adenoviral transduction efficiency was >90% for both neuronal populations, and that the expression of aSyn variants does not result in toxicity to MAP2^+^/TH^−^ neurons in this model [58]). We found that cultures expressing A30P or G51D displayed a significant loss of TH^+^ neurons relative to untransduced cultures or cultures treated with LacZ virus (Fig. 4a, b). Further analysis of the same cultures revealed that surviving TH^+^ neurons in A30P- or G51D-expressing cultures exhibited a significant reduction in neurite lengths compared to TH^+^ neurons in untransduced or β-gal-expressing cultures (Fig. 4c−e). Cultures co-expressing A30P or G51D plus WT ENSA exhibited markedly greater TH^+^ cell viability (Fig. 4a, b) and neurite lengths (Fig. 4c-e) compared to cultures expressing A30P or G51D alone or co-expressing mutant aSyn and β-gal. In contrast, expression of the S109E ENSA variant at a level similar to that of WT ENSA (Additional file 1: Figure S4) failed to rescue dopaminergic cell loss and neurite retraction in A30P- or G51D-expressing cultures (Fig. 4a−e). No changes in neurite lengths were observed in midbrain cultures expressing WT ENSA or S109E in the absence of aSyn (Additional file 1: Figure S5). Together, these results indicated that WT ENSA (but not the S109E variant) alleviated aSyn dopaminergic neurotoxicity in a primary cell culture model of PD.

### ENSA levels in the brains of human synucleinopathy patients

**Fig. 5:**
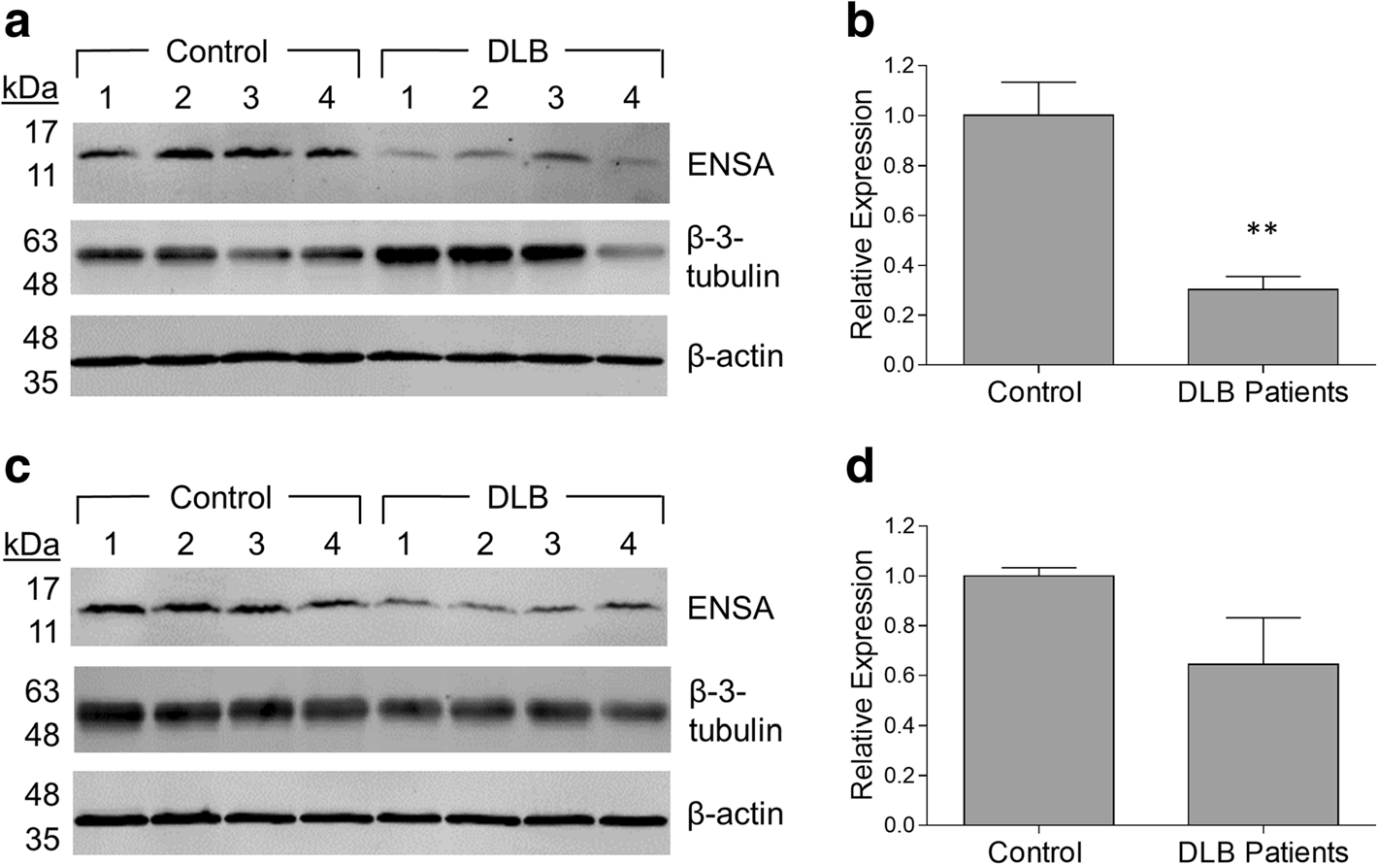
ENSA expression levels are reduced in the cortex of DLB patients. Tissue samples consisted of the frontal cortex region of DLB patients and aged-matched controls from two cohorts, provided by GIE Neuro-CEB (**a**, **b**) and Massachusetts General Hospital (**c**, **d**). The tissues were homogenized and examined for ENSA expression via Western blotting. (**a**, **c**) Images of Western blots probed with a primary antibody specific for human ENSA (upper panel), β-3-tubulin (middle panel), or β-actin (lower panel). (**b**, **d**) Bar graphs showing relative ENSA expression levels determined via densitometric analysis of Western blots for the individual cohorts. ENSA band intensities were normalized to the corresponding β-3-tubulin signals and divided by the mean value obtained for the control samples. The data are presented as the mean ± SEM, *n* = 4, **p < 0.01, two-tailed Student’s t-test

**Fig. 6:**
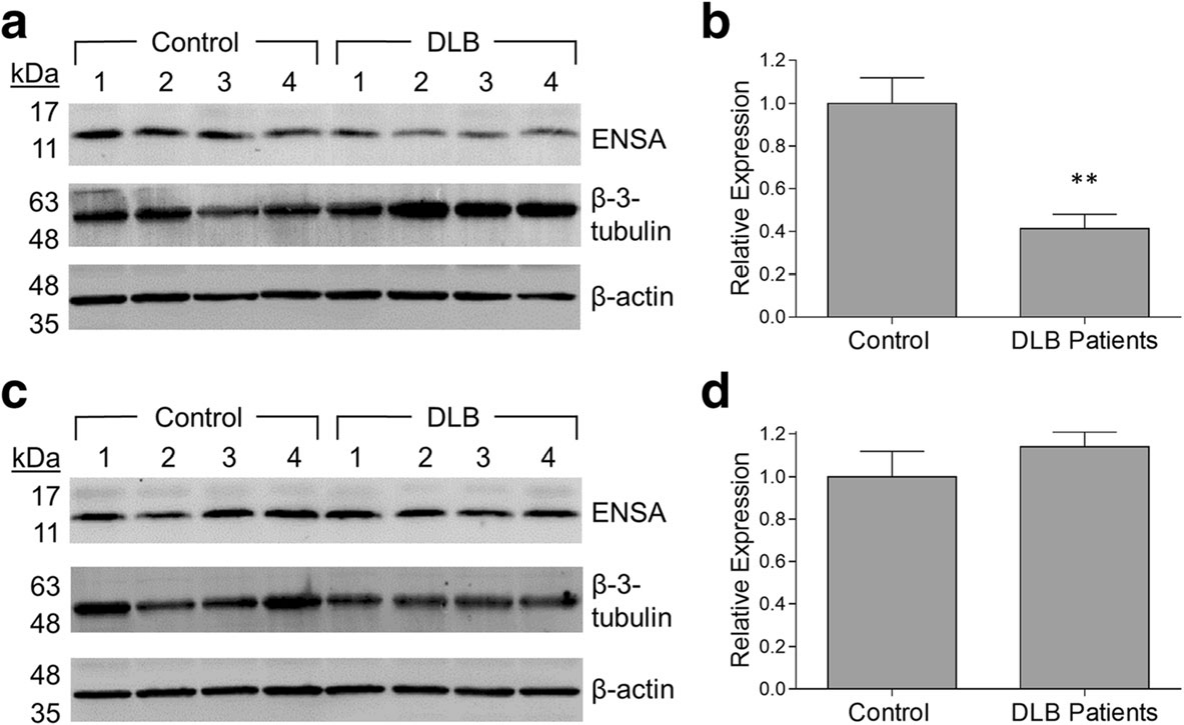
ENSA expression levels are reduced in the cingulate gyrus region of a subset of DLB patients. Tissue samples consisted of the cingulate gyrus region of DLB patients and aged-matched controls from two cohorts, provided by GIE Neuro-CEB (**a**, **b**) and Massachusetts General Hospital (**c**, **d**). The tissues were homogenized and examined for ENSA expression via Western blotting. (**a**, **c**) Images of Western blots probed with a primary antibody specific for human ENSA (upper panel), β-3-tubulin (middle panel), or β-actin (lower panel). (**b**, **d**) Bar graphs showing relative ENSA expression levels determined via densitometric analysis of Western blots for the individual cohorts. ENSA band intensities were normalized to the corresponding β-3-tubulin signals and divided by the mean value obtained for the control samples. The data are presented as the mean ± SEM, *n* = 4, **p < 0.01, two-tailed Student’s t-test

ENSA was previously shown to be down-regulated in the brains of patients with AD or Down syndrome [30, 31]. Accordingly, we hypothesized that ENSA levels might be reduced in the brains of individuals with synucleinopathy diseases, leading to a decrease in ENSA-mediated neuroprotection that could contribute to aSyn neuropathology in these disorders. To address this hypothesis, homogenates were prepared from the frontal cortex and cingulate gyrus regions of patients with a confirmed neuropathological diagnosis of DLB and individuals with no obvious neuropathology, and ENSA levels were determined via Western blot analysis. We chose to analyze samples from the frontal cortex and cingulate gyrus because both of these brain regions show evidence of aSyn neuropathology in the brains of DLB patients [21, 55]. The brain samples were prepared from two cohorts, referred to here as the ‘French’ and ‘MGH’ cohorts (Table 1). ENSA was detected in each sample as a single band corresponding to a protein with a molecular weight of ~15 kDa (Fig. 5a, c; Fig. 6a, c). ENSA band intensities determined via densitometric analysis were normalized to the respective intensities obtained for the neuronal marker β-3-tubulin to account for potential neuronal cell loss in the patient samples. The results revealed a reduction in the mean ENSA level in cortical samples of DLB patients, relative to age-matched controls, of ~70% in the French cohort (Fig. 5b; *p* < 0.01). Similarly, a strong trend towards such a reduction, resulting in a decrease in the mean ENSA level of ~40%, was observed in the MGH cohort (Fig. 5d). Analysis of homogenates prepared from the cingulate gyrus of DLB patients revealed a reduction in the mean ENSA level of ~60% relative to controls in the French cohort (Fig. 6a, b; *p* < 0.01), but no difference in the mean ENSA level between patients and controls in the MGH cohort (Fig. 6c, d). In an additional experiment, we observed a reduction in the mean ENSA level of ~50% in the *substantia nigra* region of PD patients versus non-diseased individuals, although this trend did not reach significance (Additional file 1: Figure S6a, b; Additional file 1: Table S3). Collectively, these results suggest that ENSA down-regulation could play a role in aSyn neuropathology in the brains of synucleinopathy patients.

**Table 1:** Summary of demographic information for donors of cortical and cingulate gyrus samples^a^

Case	Age (y)	Sex	Braak stage	PMI (h)	Neuropathological diagnosis
GIE Neuro-CEB BB-0033-00011 (France)					
CTRL-1	79	M	n/a	nd	control
CTRL-2	73	M	n/a	10	control
CTRL-3	78	M	n/a	23	control
CTRL-4	60	F	n/a	28	control
DLB-1	74	M	nd	21	DLB
DLB-2	83	F	nd	12	DLB
DLB-3	81	M	6	nd	DLB
DLB-4	85	M	3	21	DLB
Massachusetts General Hospital (MGH)					
CTRL-1	94	M	n/a	17	control
CTRL-2	54	M	n/a	6	control
CTRL-3	58	F	n/a	18	control
CTRL-4	92	M	n/a	nd	control
DLB-1	62	M	5	<24	DLB
DLB-2	61	M	6	20	DLB
DLB-3	65	F	6	10	DLB
DLB-4	80	F	6	nd	DLB

*Abbreviations*: *PMI* post-mortem interval, *M* male, *F* female, *n/a* not applicable, *nd* not determined

^a^Brain samples used for ENSA expression analysis were obtained from DLB patients and neuropathologically normal (control) individuals from two cohorts. The average age was 72.5 y (France) and 74.5 y (MGH) for control individuals, and 80.8 y (France) and 67.0 y (MGH) for DLB patients

## Discussion

### WT ENSA (but not the S109E variant) interferes with membrane-induced aSyn aggregation

Previous findings reported by our group and others revealed that aSyn undergoes accelerated aggregation in the presence of phospholipid membranes [20, 37], apparently by adopting an exposed, lipid-bound α-helical structure that favors the formation of β-sheet-rich assemblies on the membrane surface [4, 6, 11, 58]. Here, we confirmed our earlier results by showing that high molecular-weight, SDS-resistant forms of A30P or G51D were present in the membrane fraction of aSyn/SUV mixtures recovered by lipid flotation. These data provide direct evidence that membrane-bound aSyn oligomers were formed upon incubation of the protein with SUVs. The formation of membrane-associated aSyn oligomers could occur via two mechanisms: (i) the protein could undergo self-assembly at the membrane surface; or (ii) the protein could form oligomers in solution and subsequently bind to the membrane. Two observations are inconsistent with the second of these mechanisms. First, aSyn does not form oligomers in the absence of lipids under the incubation conditions described here (Griggs et al., unpublished observations). Second, we find that the rate at which a given aSyn variant forms oligomers in the presence of SUVs correlates poorly with the variant’s intrinsic propensity to undergo aggregation in the absence of lipids [58].

We hypothesized that ENSA, a protein that interacts selectively with membrane-bound aSyn [57], would inhibit lipid-induced aSyn self-assembly by binding to aSyn at the membrane surface, thereby disrupting contacts among exposed aSyn conformers leading to aggregate formation. Consistent with this hypothesis, we observed a marked reduction in levels of aSyn aggregates in A30P- or G51D-lipid mixtures incubated in the presence of WT ENSA. Similar amounts of membrane-associated aSyn were detected in A30P- or G51D-SUV mixtures incubated with or without ENSA under conditions suitable for aSyn binding but not aggregation, suggesting that ENSA had no major impact on the affinity of aSyn for phospholipids. Our observation that a sub-stoichiometric amount of ENSA (relative to aSyn) was sufficient to alleviate aggregation presumably reflects the fact that only a fraction of the total aSyn protein was bound to the membrane at the lipid:aSyn ratio used in these experiments [58]. Importantly, we found that introduction of the S109E mutation, which mimics PKA phosphorylation of ENSA at the highly conserved S109 residue [24] and prevents the interaction of ENSA with micelle-bound aSyn [7], completely abrogated the inhibitory effect of ENSA on membrane-induced aSyn aggregation. This result strongly suggests that WT ENSA interferes with aSyn self-assembly at the membrane surface via a direct interaction with lipid-bound aSyn.

Another key result of our study was the observation that ENSA has no impact on the ability of aSyn to form amyloid-like fibrils in the absence of vesicles. This finding is consistent with previous data showing that ENSA and aSyn only bind to each other at the membrane surface, apparently because both are natively unfolded in the absence of phospholipids but adopt an α-helical structure compatible with their interaction upon membrane binding [57]. The fact that ENSA has no impact on aSyn fibrillization in the absence of lipids suggests that the inhibitory effect of ENSA on aSyn aggregation in the presence of SUVs must be related to its interaction with aSyn at the membrane surface. This evidence further supports the idea that aSyn actively oligomerizes on the membrane when incubated in the presence of SUVs under the conditions described in this study.

Collectively, our data show for the first time that a protein can inhibit membrane-induced aSyn aggregation via an apparent chaperone-like activity at the membrane surface. NMR chemical shift data revealed that the binding of ENSA to aSyn in the presence of SDS micelles primarily involves ENSA residues 28–36 and 88–97, with a smaller contribution from residues near S109 [7], and aSyn residues 19–30 and 58–74 [57]. Because residues 58–74 of aSyn are located in the dissociated, aggregation-prone region of exposed, lipid-bound conformers [6, 58], we infer that ENSA could inhibit membrane-induced aSyn self-assembly by: (i) disrupting interactions involving the dissociated region of neighboring exposed conformers, and/or (ii) promoting binding of the dissociated region to the bilayer, thereby stabilizing a more hidden form of membrane-bound aSyn.

### WT ENSA (but not the S109E variant) interferes with aSyn-mediated vesicle disruption

An important outcome of our study was the finding that aSyn variants trigger vesicle permeabilization under conditions that promote membrane-induced aggregation. To monitor vesicle disruption, we developed a novel fluorescence-based assay that reported on the time-dependent loss of membrane integrity of calcein-loaded SUVs incubated with aSyn variants initially added in monomeric form [3, 58]. This assay involves the extended incubation of aSyn-SUV mixtures under conditions shown to promote aggregation at the membrane surface (Fig. 1) and is thus distinct from previously reported dye leakage assays designed to monitor the permeabilization of phospholipid vesicles immediately upon the addition of aSyn oligomers prepared in the absence of membranes [52, 54]. Using this assay, which served as a direct measure of the physiological effect of aSyn aggregation at the membrane surface, we showed that A30P and G51D both triggered membrane leakage to an extent consistent with the degree of aggregation at each time point [58]. In contrast, the non-familial mutant A29E, which has a weak propensity to undergo membrane-induced aggregation [58], failed to elicit vesicle disruption, strongly suggesting that aSyn-mediated membrane permeabilization is closely related (or even coupled) to aSyn self-assembly at the membrane surface. Oligomeric aSyn prepared in the absence of membranes was previously shown to permeabilize low-diameter vesicles composed of anionic lipids, but not a mixture of anionic and zwitterionic lipids [54]. Consistent with these findings, pre-formed aSyn oligomers were inactive in our assay using PG:PC SUVs. Together, these observations suggest that the vesicle permeabilization observed in aSyn-SUV mixtures in this study occurs as a result of aSyn aggregation at the membrane surface, possibly via a mechanism involving lipid extraction from the bilayer and membrane thinning [11, 37, 44]. This evidence further supports the idea that aSyn forms aggregates on the membrane surface under the experimental conditions described here.

Importantly, we found that WT ENSA interfered with membrane disruption triggered by A30P or G51D. This inhibitory effect was highly dose-dependent, with a significant reduction in permeabilization observed at an ENSA:aSyn ratio as low as 1:4 (mol/mol) – again implying that only a fraction of the total aSyn protein was bound to the membrane under these experimental conditions. In contrast to WT ENSA, the S109E variant had no effect on A30P- or G51D-induced membrane permeabilization, suggesting that WT ENSA attenuated aSyn-mediated vesicle disruption by binding directly to membrane-associated aSyn. Our observation that WT ENSA (but not S109E) inhibited both membrane-induced aSyn aggregation and vesicle disruption underscores the idea that these phenomena are closely related. Accordingly, ENSA-aSyn interactions responsible for disrupting aSyn self-assembly at the membrane surface could also be involved in suppressing membrane permeabilization. To our knowledge, this is the first report of a protein with the ability to inhibit aSyn-mediated vesicle disruption via an apparent chaperone-like activity at the membrane surface.

### WT ENSA (but not the S109E variant) interferes with aSyn neurotoxicity

In a previous report, we showed that A30P and G51D had a greater propensity than WT aSyn to undergo lipid-induced self-assembly at the membrane surface and elicit dopaminergic cell death in primary midbrain cultures [58]. Here, we showed that the neurotoxicity of both aSyn variants was alleviated by co-expressing WT ENSA, but not the S109E variant. Based on the evidence reported here that WT ENSA, but not S109E, interferes with membrane-induced aSyn aggregation and aSyn-mediated vesicle disruption via a chaperone-like activity, we infer that the ability of ENSA to inhibit these pathological effects of aSyn at the membrane surface plays a central role in its neuroprotective activity.

Although A30P and G51D are rare familial aSyn mutants, the effects of ENSA on the aggregation propensity, permeabilization activity, and neurotoxicity of these variants as reported here are likely relevant to sporadic PD and other synucleinopathy disorders including DLB because there is evidence that the neuropathological features elicited by WT aSyn in the brains of synucleinopathy patients overlap with those caused by aSyn substitution mutants in familial PD [29, 46]. In addition, PD patients with a duplication or triplication of the aSyn gene have similar clinical and neuropathological phenotypes compared to patients with familial aSyn substitutions, suggesting that the neurotoxicity of WT aSyn is related to, but intrinsically more moderate than, that of the familial substitution mutants. Consistent with this idea, membrane-bound WT aSyn has been shown to adopt a more exposed, aggregation-prone conformation at higher protein-to-lipid ratios [6]. Moroever, by analogy with the effects of aSyn mutations, membrane perturbations including lipid oxidation and changes in lipid composition observed in synucleinopathy patients [15, 47] would be predicted to favor the self-assembly of membrane-bound WT aSyn [58]. Under these conditions, WT aSyn could potentially elicit similar neuropathological effects as A30P or G51D. Based on this reasoning and evidence that ENSA interacts with WT aSyn [7, 57], we predict that ENSA should alleviate aSyn-mediated neurodegeneration in the brains of patients not only with familial PD, but also with sporadic PD and other synucleinopathy disorders. However, this idea was not tested here because of the limited neurotoxicity of WT aSyn in our primary cell culture model [58].

### A decrease in ENSA levels is observed in the brains of synucleinopathy patients

ENSA is a member of a highly conserved family of cAMP-regulated phosphoproteins that includes ARPP16/19, a protein that was also found to interact with membrane-bound aSyn [57]. Members of this family have been most extensively characterized in terms of their roles in cell division related to cancer biology [18]. However, these proteins are also highly expressed in rat brain tissue, particularly in neurons of the frontal cortex and the basal ganglia [17, 18]. ENSA has been found to be down-regulated in the brains of AD patients and individuals with Down syndrome [30, 31]. Here, we observed a decrease in ENSA levels in DLB cortex (or a strong trend towards such a decrease) in two independent cohorts. ENSA levels were also reduced in the cingulate gyrus of DLB patients in one of two cohorts examined. The fact that the cingulate gyrus samples of DLB patients from the French cohort, but not the MGH cohort, showed evidence of ENSA down-regulation may reflect differences in disease progression between the two sets of patients. Finally, we observed a pronounced trend towards a decrease in ENSA levels in the *substantia nigra* of PD patients.

The fact that patients with the A30P or G51D mutation are characterized by aSyn neuropathology similar to that in the brains of DLB patients [29, 38, 46] implies that the two mutants and WT aSyn elicit neurodegeneration via similar mechanisms. In turn, we infer that the inhibitory effects of ENSA on the aggregation propensity, permeabilization activity, and neurotoxicity of A30P or G51D may be relevant to the effects of ENSA down-regulation on WT aSyn neuropathology in the brains of DLB patients. Collectively, our results suggest that (i) ENSA down-regulation could favor aSyn aggregation and neurotoxicity in synucleinopathy diseases; and (ii) up-regulation of ENSA expression in the brains of synucleinopathy patients, at least to basal levels, could be a viable neuroprotective strategy.

### Stabilization of membrane-bound amyloidogenic protein is a neuroprotective strategy to treat synucleinopathies and other protein misfolding diseases

Our observation that WT ENSA blocked aSyn self-assembly at the membrane surface and alleviated aSyn-mediated dopaminergic cell death, but had no effect on aSyn fibrillization in the absence of vesicles, further highlights the critical role of membrane-induced aSyn aggregation in aSyn neurotoxicity. The idea that lipid-induced aSyn self-assembly plays a key role in PD pathogenesis is also supported by evidence that non-membrane-bound aSyn exists in a compact, aggregation-resistant state in mammalian cells, with the central hydrophobic domain shielded from interactions with cytosolic components [51]. In turn, this finding implies that membrane binding could serve as a key trigger for aSyn aggregation by inducing exposure of the hydrophobic domain.

Collectively, these observations suggest that selectively targeting membrane-bound aSyn could be a viable therapeutic strategy for PD and other synucleinopathy disorders. Previous results obtained by our group and others revealed that up-regulating molecular chaperones that interact with aSyn in the absence of lipid membranes, including Hsp27, Hsp70, and DJ-1, could result in attenuation of aSyn neurotoxicity [32, 39, 40]. Here we propose a different neuroprotective strategy involving the up-regulation of proteins that bind specifically to membrane-associated aSyn, thereby stabilizing a more hidden aSyn structure and/or interfering with interactions among exposed conformers at the membrane surface. In addition to ENSA, other proteins that have been shown to bind specifically to membrane-associated aSyn, including PLD2 [26], synaptobrevin-2 [8], and Rab3a [10], could potentially be candidates for such a membrane-targeted chaperone strategy.

A number of other amyloidogenic polypeptides have been found to undergo lipid-induced aggregation coupled to membrane permeabilization, including islet amyloid polypeptide (IAPP) involved in diabetes [2, 19, 33] and Aβ peptide associated with AD neuropathology [14]. The results presented here imply that protein binding partners could also interfere with the aggregation of these polypeptides at the membrane surface. In support of this idea, insulin has been shown to interfere with lipid-induced IAPP self-assembly, although this inhibitory effect is distinct from that of ENSA on membrane-induced aSyn aggregation because it involves the binding of insulin to IAPP fibril ends rather than the membrane and is markedly greater in the absence than in the presence of lipids [34]. In other studies, peptidomimetics and small molecules that interfered with membrane-induced IAPP aggregation were reported to attenuate IAPP cytotoxicity [23, 35]. These observations and the findings presented herein strongly suggest that stabilizing aggregation-resistant forms of membrane-bound amyloidogenic polypeptides by promoting interactions with their binding partners could be a viable therapeutic strategy not only for synucleinopathy disorders, but also for a broader array of protein misfolding diseases.

## Conclusions

In this study, we observed that the interaction of ENSA with aSyn at the surface of phospholipid vesicles led to a decrease in membrane-induced aSyn aggregation and aSyn-mediated vesicle permeabilization. In addition, ENSA over-expression led to a decrease in aSyn neurotoxicity in primary midbrain cultures, and ENSA was found to be down-regulated in the brains of synucleinopathy patients. These findings suggest that interfering with aSyn self-assembly at membrane surfaces could slow neurodegeneration in PD and other synucleinopathies, and they provide a strong rationale for examining neuroprotective effects of ENSA in animal models of these disorders.

## Additional file

Additional file 1: Figures S1-S6, Tables S1-S3.
**Figure S1.** ENSA does not have a pronounced effect on the binding of aSyn to phospholipid membranes. **Figure S2.** A29E aSyn fails to elicit membrane disruption. **Figure S3.** ENSA exhibits no membrane disruption activity on its own. **Figure S4.** Results of Western blot analysis showing equal expression levels of WT ENSA and S109E in primary midbrain cultures. **Figure S5.** WT ENSA and S109E do not elicit neurotoxicity when expressed alone or in combination with β-gal. **Figure S6.** Western blot image showing a trend towards reduced ENSA expression levels in the substantia nigra region of PD patients versus non-diseased individuals. **Table S1.** *P* values for vesicle permeabilization data in Fig. 3a and b. **Table S2.** *P* values for vesicle permeabilization data in Fig. 3c and d. **Table S3.** Summary of demographic information for donors of substantia nigra samples. (DOCX 421 kb)
