# Supplementary material for: Endosulfine-alpha inhibits membrane-induced α-synuclein aggregation and protects against α-synuclein neurotoxicity
Source: Acta Neuropathol Commun. 2017 Jan 10;5:3. doi: 10.1186/s40478-016-0403-7 (PMC5223451; doi:10.1186/s40478-016-0403-7)
Supplement: Additional file 1: Figures S1-S6, Tables S1-S3. — Figure S1. ENSA does not have a pronounced effect on the binding of aSyn to phospholipid membranes. Figure S2. A29E aSyn fails to elicit membrane disruption. Figure S3. ENSA exhibits no membrane disruption activity on its own. Figure S4. Results of Western blot analysis showing equal expression levels of WT ENSA and S109E in primary midbrain cultures. Figure S5. WT ENSA and S109E do not elicit neurotoxicity when expressed alone or in combination with β-gal. Figure S6. Western blot image showing a trend towards reduced ENSA expression levels in the substantia nigra region of PD patients versus non-diseased individuals. Table S1. P values for vesicle permeabilization data in Fig. 3a and b. Table S2. P values for vesicle permeabilization data in Fig. 3c and d. Table S3. Summary of demographic information for donors of substantia nigra samples. (DOCX 421 kb) [file 40478_2016_403_MOESM1_ESM.docx]

**Additional File**

Endosulfine-alpha inhibits membrane-induced α-synuclein aggregation and protects against α-synuclein neurotoxicity

Daniel Ysselstein^1,2^, Benjamin Dehay^3,4^, Isabel M. Costantino^5^, George P. McCabe^6^, Matthew P. Frosch^5^, Julia M. George^7^, Erwan Bezard^3,4^, Jean-Christophe Rochet^1,2,^*

^1^Department of Medicinal Chemistry and Molecular Pharmacology, Purdue University, West Lafayette, IN, USA

^2^Purdue Institute for Integrative Neuroscience, Purdue University, West Lafayette, IN, USA

^3^Université de Bordeaux, Institut des Maladies Neurodégénératives, UMR 5293, Bordeaux, France

^4^CNRS, Institut des Maladies Neurodégénératives, UMR 5293, Bordeaux, France

^5^Department of Neurology, Massachusetts Alzheimer's Disease Research Center, Massachusetts General Hospital, Charlestown, MA, USA

^6^Department of Statistics, Purdue University, West Lafayette, IN, USA

^7^Department of Biological and Experimental Psychology, School of Biological and Chemical Sciences, Queen Mary University of London, London, UK

*To whom correspondence may be addressed: Email: jrochet@purdue.edu

**
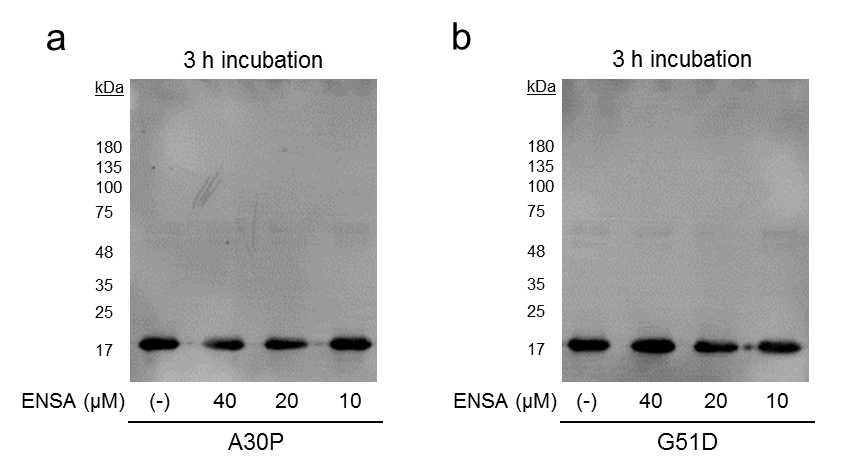
Figure S1. ENSA does not have a pronounced effect on the binding of aSyn to phospholipid membranes.** A mixture of A30P (A) or G51D (B) (40 μM of each) and SUVs was incubated in the absence (-) or presence of WT ENSA (10-40 μM) for 3 h. Membrane fractions were isolated and analyzed via Western blotting.

**Figure S2. A29E aSyn fails to elicit membrane disruption.** Calcein-loaded SUVs were incubated in the absence (‘control’) or presence of A30P, G51D, or A29E (40 µM of each). In a previous study we showed that A29E, in contrast to A30P and G51D, lacks the ability to undergo membrane-induced aggregation [58]. Calcein release was monitored via fluorescence measurements at an excitation wavelength of 485 nm and an emission wavelength of 515 nm. The data are presented as % dye release versus time, with 100% release determined as the signal obtained from vesicles treated with Triton X-100 (n = 2, control and A29E; n = 1, A30P and G51D).

**Figure S3. ENSA exhibits no membrane disruption activity on its own.** Calcein-loaded SUVs were incubated in the absence (‘dye alone’) or presence of ENSA (10-40 µM). Calcein release was monitored via fluorescence measurements at an excitation wavelength of 485 nm and an emission wavelength of 515 nm. The data are presented as % dye release versus time, with 100% release determined as the signal obtained from vesicles treated with Triton X-100. Mean ± SEM, n = 3.


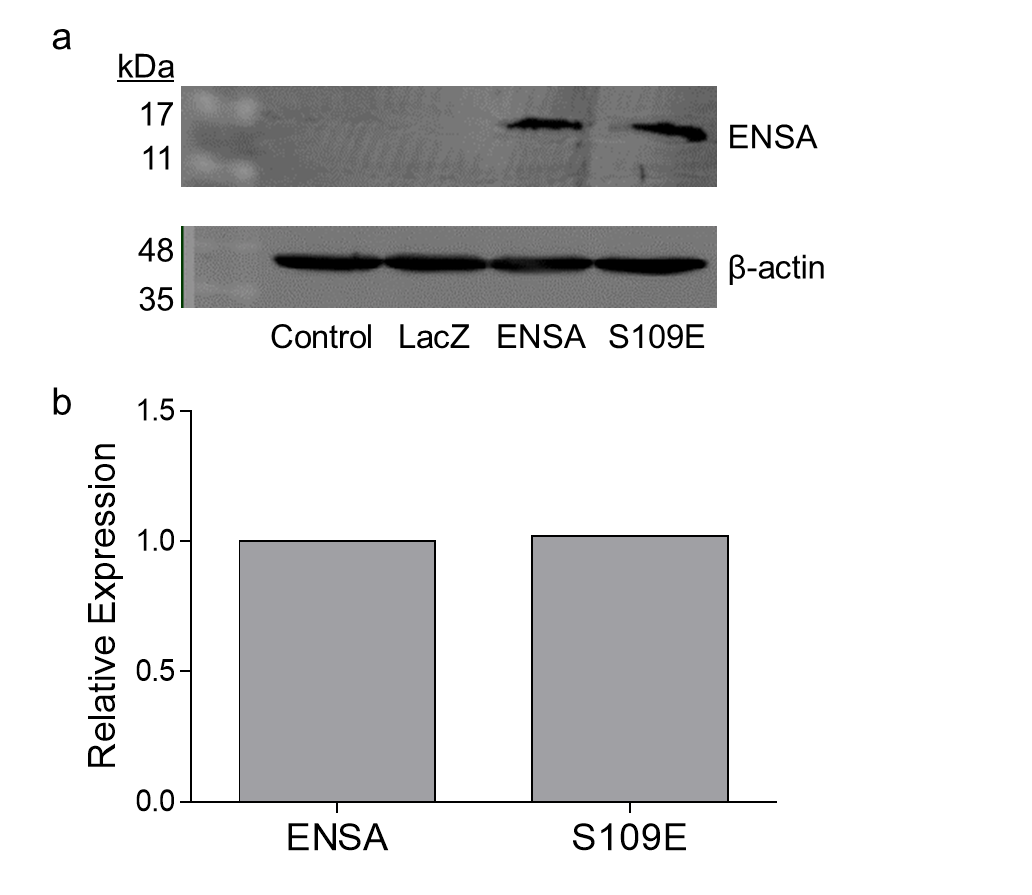
**Figure S4. Results of Western blot analysis showing equal expression levels of WT ENSA and S109E in primary midbrain cultures.** Lysates from cultures incubated in the absence (‘control’) or presence of adenovirus encoding β-gal (‘LacZ’), WT ENSA, or S109E at an MOI of 5 were analyzed via Western blotting. (**a**) Images of Western blots probed with a primary antibody specific for human ENSA (upper panel) or β-actin as a loading control (lower panel). The blot in the upper panel reveals a single band at ~15 kDa in lanes loaded with lysates of cells expressing ENSA, but not β-gal, confirming the specificity of the anti-ENSA antibody for the human protein. (**b**) Bar graph showing relative ENSA expression levels determined via densitometric analysis of the Western blot. ENSA band intensities were normalized to the corresponding β-actin signals and divided by the value obtained for cells expressing WT ENSA.

**
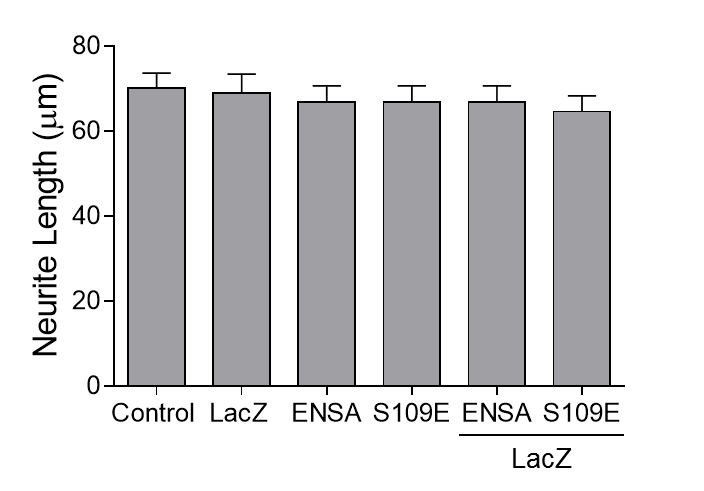
Figure S5. WT ENSA and S109E do not elicit neurotoxicity when expressed alone or in combination with β-gal.** Primary midbrain cultures were transduced with adenoviruses encoding LacZ, WT ENSA, S109E, LacZ plus WT ENSA, or LacZ plus S109E at MOIs equivalent to those used in the experiment described in Fig. 4. Additional cultures were untransduced (‘control’). The cells were fixed, stained with antibodies specific for MAP2 and TH, and scored for neurite lengths. The data are presented as the mean ± SEM (n = 1).

**
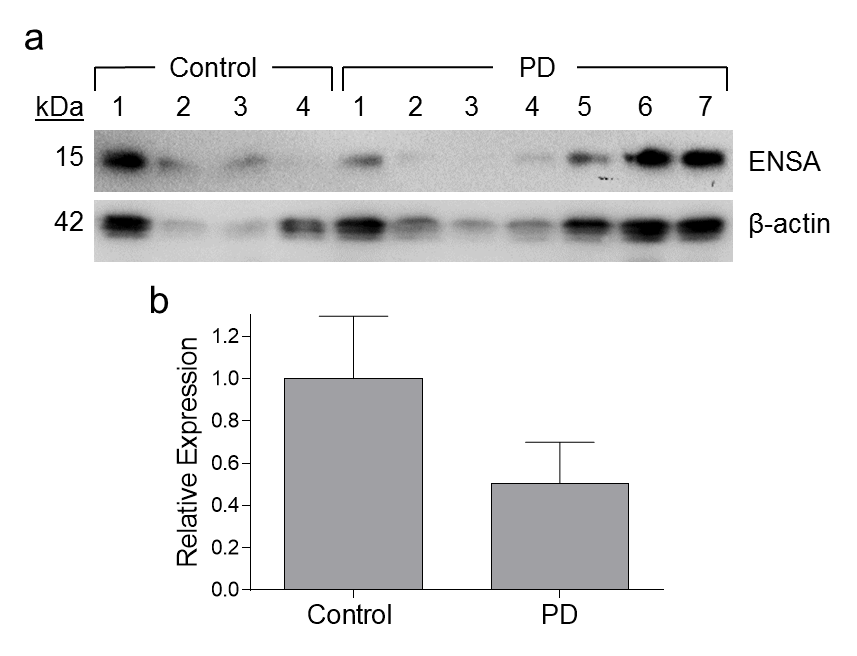
**

**Figure S6. Western blot image showing a trend towards reduced ENSA expression levels in the *substantia nigra* region of PD patients versus non-diseased individuals.** Tissue samples consisted of the *substantia nigra* region of PD patients and age-matched controls, provided by GIE Neuro-CEB. The tissues were homogenized and examined for ENSA expression via Western blotting. (**a**) Images of a Western blot probed with a primary antibody specific for human ENSA (upper panel) or β-actin (lower panel) as a loading control. (**b**) Bar graph showing relative ENSA expression levels determined via densitometric analysis of the Western blot. ENSA band intensities were normalized to the corresponding β-actin signals and divided by the mean value obtained for control samples. The data are presented as the mean ± SEM, n = 4 (controls) or n = 7 (patients). Although there is variation in the β-actin band intensities among the different samples, this variation does not affect the interpretation of the data because the ENSA band intensities were normalized to the corresponding β-actin signals. Moreover, the ~50% down-regulation of ENSA shown in panel B cannot be attributed to nigral neurodegeneration because the total neuronal loss in PD *substantia nigra* relative to controls is much less than 50%.

Table S1: *P* values for vesicle permeabilization data in Fig. 3 a and b ^a^

| Comparison | Time (h) | | | | |
| --- | --- | --- | --- | --- | --- |
|  | 24 | 48 | 72 | 96 | 120 |
| Dye vs. A30P | **** | **** | **** | **** | **** |
| Dye vs. A30P ENSA 10 µM | **** | **** | **** | **** | **** |
| Dye vs. A30P ENSA 20 µM | **** | **** | **** | **** | **** |
| Dye vs. A30P ENSA 40 µM | *** | *** | **** | **** | **** |
| A30P vs. A30P ENSA 10 µM | ns | ns | *** | **** | **** |
| A30P vs. A30P ENSA 20 µM | ns | ** | **** | **** | **** |
| A30P vs. A30P ENSA 40 µM | * | **** | **** | **** | **** |
| A30P ENSA 10 µM vs. A30P ENSA 20 µM | ns | ns | ns | ns | ns |
| A30P ENSA 10 µM vs. A30P ENSA 40 µM | ns | ** | **** | **** | **** |
| A30P ENSA 20 µM vs. A30P ENSA 40 µM | ns | ns | ** | *** | ** |

| Comparison | Time (h) | | | | |
| --- | --- | --- | --- | --- | --- |
|  | 24 | 48 | 72 | 96 | 120 |
| Dye vs. G51D | **** | **** | **** | **** | **** |
| Dye vs. G51D ENSA 10 µM | *** | **** | **** | **** | **** |
| Dye vs. G51D ENSA 20 µM | **** | **** | **** | **** | **** |
| Dye vs. G51D ENSA 40 µM | ** | *** | **** | **** | **** |
| G51D vs. G51D ENSA 10 µM | ns | ns | *** | **** | *** |
| G51D vs. G51D ENSA 20 µM | ns | ** | **** | **** | **** |
| G51D vs. G51D ENSA 40 µM | * | **** | **** | **** | **** |
| G51D ENSA 10 µM vs. G51D ENSA 20 µM | ns | ns | ns | ns | * |
| G51D ENSA 10 µM vs. G51D ENSA 40 µM | ns | ns | *** | ** | **** |
| G51D ENSA 20 µM vs. G51D ENSA 40 µM | ns | ns | ns | ns | ns |

^a^ Two-way ANOVA, *p<0.05, **p<0.01, ***p<0.001, ****p<0.0001

Table S2: *P* values for vesicle permeabilization data in Fig. 3 c and d ^a^

| Comparison | Time (h) | | | | |
| --- | --- | --- | --- | --- | --- |
|  | 24 | 48 | 72 | 96 | 120 |
| Dye vs. A30P Alone | **** | **** | **** | **** | **** |
| Dye vs. A30P ENSA | * | *** | **** | **** | **** |
| Dye vs. A30P S109E | **** | **** | **** | **** | **** |
| A30P vs. A30P ENSA | ** | **** | **** | **** | **** |
| A30P vs. A30P S109E | ns | ns | ns | ns | ns |
| A30P ENSA vs. A30P S109E | * | **** | **** | **** | **** |

| Comparison | Time (h) | | | | |
| --- | --- | --- | --- | --- | --- |
|  | 24 | 48 | 72 | 96 | 120 |
| Dye vs. G51D | **** | **** | **** | **** | **** |
| Dye vs. G51D ENSA | * | *** | **** | **** | **** |
| Dye vs. G51D S109E | **** | **** | **** | **** | **** |
| G51D vs. G51D ENSA | * | **** | **** | **** | **** |
| G51D vs. G51D S109E | ns | ns | ns | ns | ns |
| G51D ENSA vs. G51D S109E | ns | **** | **** | **** | **** |

^a^ Two-way ANOVA, *p<0.05, **p<0.01, ***p<0.001, ****p<0.0001

Table S3. Summary of demographic information for donors of *substantia nigra* samples^a^

| GIE Neuro-CEB BB-0033-00011 (France) | | | | |
| --- | --- | --- | --- | --- |
| Case | Age (y) | Sex | PMI (h) | Neuropathological diagnosis |
| CTRL-1 | 87 | M | 7 | control |
| CTRL-2 | 31 | M | 17.5 | control |
| CTRL-3 | 82 | M | 2.5 | control |
| CTRL-4 | 61 | M | 7 | control |
| PD-1 | 76 | M | 2 | PD |
| PD-2 | 76 | M | 3 | PD |
| PD-3 | 75 | M | 10 | PD |
| PD-4 | 85 | F | 15 | PD |
| PD-5 | 74 | M | 30 | PD |
| PD-6 | 76 | F | 20 | PD |
| PD-7 | 81 | M | 28 | PD |

^a^Brain samples used for ENSA expression analysis were obtained from PD patients and neuropathologically normal (CTRL) individuals. The average age was 65.2 y and 77.6 y for control individuals and patients, respectively. Abbreviations: PMI, post-mortem interval; M, male; F, female.
